# Signaling Mechanism for Modulation by GLP-1 and Exendin-4 of GABA Receptors on Rat Retinal Ganglion Cells

**DOI:** 10.1007/s12264-022-00826-9

**Published:** 2022-03-12

**Authors:** Tao Zhang, Hang-Ze Ruan, Yong-Chen Wang, Yu-Qi Shao, Wei Zhou, Shi-Jun Weng, Yong-Mei Zhong

**Affiliations:** State Key Laboratory of Medical Neurobiology and MOE Frontiers Center for Brain Science, Institutes of Brain Science, Fudan University, Shanghai, 200032 China

**Keywords:** Glucagon-like peptide-1, Exendin-4, GABA current, Retinal ganglion cells, Neuromodulation

## Abstract

Glucagon-like peptide-1 (GLP-1) is expressed in retinal neurons, but its role in the retina is largely unknown. Here, we demonstrated that GLP-1 or the GLP-1 receptor (GLP-1R; a G protein-coupled receptor) agonist exendin-4 suppressed γ-aminobutyric acid receptor (GABAR)-mediated currents through GLP-1Rs in isolated rat retinal ganglion cells (GCs). Pre-incubation with the stimulatory G protein (G_s_) inhibitor NF 449 abolished the exendin-4 effect. The exendin-4-induced suppression was mimicked by perfusion with 8-Br-cAMP (a cAMP analog), but was eliminated by the protein kinase A (PKA) inhibitor Rp-cAMP/KT-5720. The exendin-4 effect was accompanied by an increase in [Ca^2+^]_i_ of GCs through the IP_3_-sensitive pathway and was blocked in Ca^2+^-free solution. Furthermore, when the activity of calmodulin (CaM) and CaM-dependent protein kinase II (CaMKII) was inhibited, the exendin-4 effect was eliminated. Consistent with this, exendin-4 suppressed GABAR-mediated light-evoked inhibitory postsynaptic currents in GCs in rat retinal slices. These results suggest that exendin-4-induced suppression may be mediated by a distinct G_s_/cAMP-PKA/IP_3_/Ca^2+^/CaM/CaMKII signaling pathway, following the activation of GLP-1Rs.

**Supplementary Information:**

The online version of this article (10.1007/s12264-022-00826-9) contains supplementary material, which is available to authorized users.

## Introduction

Glucagon-like-peptide-1 (GLP-1) is a metabolic hormone secreted by intestinal endocrine L-cells and stimulates insulin secretion in a glucose-dependent manner [1]. GLP-1 is also produced in the brain, particularly from preproglucagon neurons, which are distributed in the solitary tract of the brain stem, and it functions as a neuropeptide [2–5]. This peptide has been implicated in modulating neuronal cell differentiation, neurite outgrowth, and performing neuroprotective functions through the activation of GLP-1 receptors (GLP-1Rs), class B sub-group G protein-coupled receptors [2, 6].

GLP-1 expression has been found in the vertebrate retina [7–9]. Specifically, GLP-1R immunoreactivity has been observed in the ganglion cell layer (GCL) in the human and rat retina [7, 10–12], and sparse staining has also been detected in the inner and outer nuclear layers of the human retina. More recently, systemic or topical administration (eye drops) of GLP-1 and GLP-1R agonists has been reported to prevent electroretinogram abnormalities and neurodegeneration of the retina in rat and mouse models of diabetes [7, 10]. However, whether GLP-1 modulates retinal information processing is still largely unknown.

In the retina, ganglion cells (GCs) are the sole output neurons that integrate inhibitory signals from amacrine cells and excitatory signals from bipolar cells and transmit the processed information to higher centers [13]. GABA receptors (GABARs) and glycine receptors mediate the inhibitory signals of amacrine cells onto GCs. It has been shown that GLP-1/the GLP-1R agonist exendin-4 increases GABA_A_R-mediated tonic currents through GLP-1R activation in rat hippocampal CA3 pyramidal neurons [14], but no data concerning the regulation of GABARs of retinal neurons by GLP-1/GLP-1R agonists are now available. In the present work, we used whole-cell patch-clamp recording techniques to explore whether GLP-1 or exendin-4 modulates GABARs of rat retinal GCs. We first demonstrated that GLP-1 or the GLP-1R agonist exendin-4 suppressed GABAR-mediated currents (GABA currents) in isolated rat retinal GCs through GLP-1R activation. By pharmacological approaches, we further showed that a distinct G_s_/cAMP-protein kinase A (PKA)/inositol 1,4,5-trisphosphate (IP_3_)/Ca^2+^/calmodulin (CaM)/CaM-dependent protein kinase II (CaMKII) signaling pathway is responsible for the exendin-4 effect on GCs. Consistent with this, we also showed that exendin-4 suppressed GABAR-mediated the light-evoked inhibitory postsynaptic currents (L-IPSCs) of GCs *via* GLP-1Rs.

## Materials and Methods

### Animals

Male Sprague–Dawley rats (15–20 days of age) were used in this study. All procedures were approved by the Animal Care and Use Committee of the Shanghai Medical College of Fudan University and were performed according to the National Institutes of Health Guide for the Care and Use of Laboratory Animals. All participants signed the written informed consent.

### Retrograde Labeling of GCs

The detailed procedure were based on previous work [15, 16]. GCs were retrogradely labeled by 20% rhodamine-labeled fluorescent latex microspheres (LumaFluor, Durham, USA).

### Preparation of Isolated GCs

As previously reported [16], GCs were isolated by enzymatic digestion of retinal tissue with papain and mechanical separation. Rhodamine-labeled GCs (15–25 μm in diameter) were used for electrophysiological recording within 2–3 h after separation.

### Whole-cell Patch-clamp Recording

Whole-cell membrane currents were recorded from rhodamine-labeled GCs using a patch amplifier (EPC9/2, HEKA Elektronik, Lambrecht, Germany) with Pulsefit 8.80 software (HEKA Elektronik). The sampling rate was set at 5 kHz and with a high-pass filter at 2 kHz .The recording pipettes (6–8 MΩ) were filled with internal solution containing (in mmol/L) CsCl 120, CaCl_2_ 1, MgCl_2_ 2, EGTA 10, HEPES 10, ATP-Mg 2, GTP-Na 0.4, NaCl 5, and phosphocreatine 10; adjusted to pH 7.25 with CsOH and osmolality to 280–300 mOsm/L with sucrose, as in our previous paper [17]. The GCs were bathed in Ringer’s that contained (in mmol/L) NaCl 145, KCl 5, CaCl_2_ 2, MgCl_2_ 1, HEPES 10, and glucose 16; pH adjusted to 7.4 with NaOH [18]. The cells were clamped at −60 mV and GABA (30 μmol/L; 5 s) was administered every 2 min to induce currents. All recordings were captured at 20–25°C.

We recorded GABAR-mediated L-IPSCs in retinal slices that were prepared as described in detail previously [19]. The slices were transferred to a recording chamber and superfused constantly with carbogen (95% O_2_/5% CO_2_)-bubbled Ames medium (Sigma-Aldrich, Inc., St. Louis, USA). The pipette solution consisted of the following (in mmol/L): TEA-Cl 10, CsCH_3_SO_3_ 120, HEPES 10, EGTA 1, CaCl_2_ 0.1, phosphocreatine 12, GTP-Na 0.5, ATP-Mg 3; pH adjusted to 7.25 with CsOH. In the GCL, we distinguished GCs from displaced amacrine cells based on cell body diameter and physiological criteria [20–22]. The GABAR-mediated L-IPSCs of GCs were recorded with a patch amplifier (MultiClamp 700B, Molecular Devices, Novato, USA). The cells were clamped at 0 mV. A mixture of synaptic blockers that contained tetrodotoxin (TTX; 1 μmol/L) and strychnine (1 μmol/L) was added to the Ames medium to block voltage-gated Na^+^ channels and glycine receptors, respectively. An LED (*λ* = 470 nm) was used to generate full-field light stimuli (0.3−0.5 μW/cm^2^, 3 s, at 60-s intervals); it was controlled by Polylite software (Mightex Systems, Pleasanton, USA) and transmitted to the retina.

### Ca^2+^ Imaging

We used the membrane permeability indicator Fura-2AM (Dojindo, Kumamoto, Japan) to assess changes in the intracellular Ca^2+^ concentration ([Ca^2+^]_i_) of isolated GCs as described previously [15]. Fluorescence images were captured on an Olympus inverted microscope equipped with a digital CCD camera (Hamamatsu Photonics, Shizuoka, Japan). We used high-speed continuous scanning monochromatic light sources (Till Photonics, Grafeling, Germany) to excite at 340 nm and 380 nm. Fluorescence intensities at 340 nm and 380 nm (F_340_ and F_380_) were measured every 1–10 s, and images were acquired using C-imaging systems (Hamamatsu Photonic). The [Ca^2+^]_i_ of the cell was proportional to the ratio of fluorescence intensity between the two images. Before an experiment, we measured the background fluorescence level and subtracted it from the obtained data.

## Chemicals

GLP-1, exendin-4, and exendin(9-39) were from AnaSpec (Fremont, USA); U73122, ryanodine, and W-7 hydrochloride were from Tocris Bioscience (Ellisville, USA). All other chemicals were from Sigma-Aldrich. The RSC-200 (Bio-Logic, Claix, France), a fast solution exchanger based on a stepping motor, was used for solution transport, with a solution exchange time being ~5 ms.

## Statistical Analysis

Data were analyzed using Pulsefit (HEKA Elektronik, Lambrecht/Pfalz, Germany), Igor 4.0 (WaveMetrics, Lake Oswego, USA), and SigmaPlot 12.0 (Systat Software, Inc., San Jose, USA). Data are shown as the mean ± SEM. Significant differences were identified by either paired Student’s *t*-test (for paired data) or one-way ANOVA with *post hoc* Tukey’s test (for multiple comparisons). For all analyses, *P* <0.05 was considered statistically significant.

## Results

### GABAR-mediated Currents in Rat GCs

**Fig. 1:**
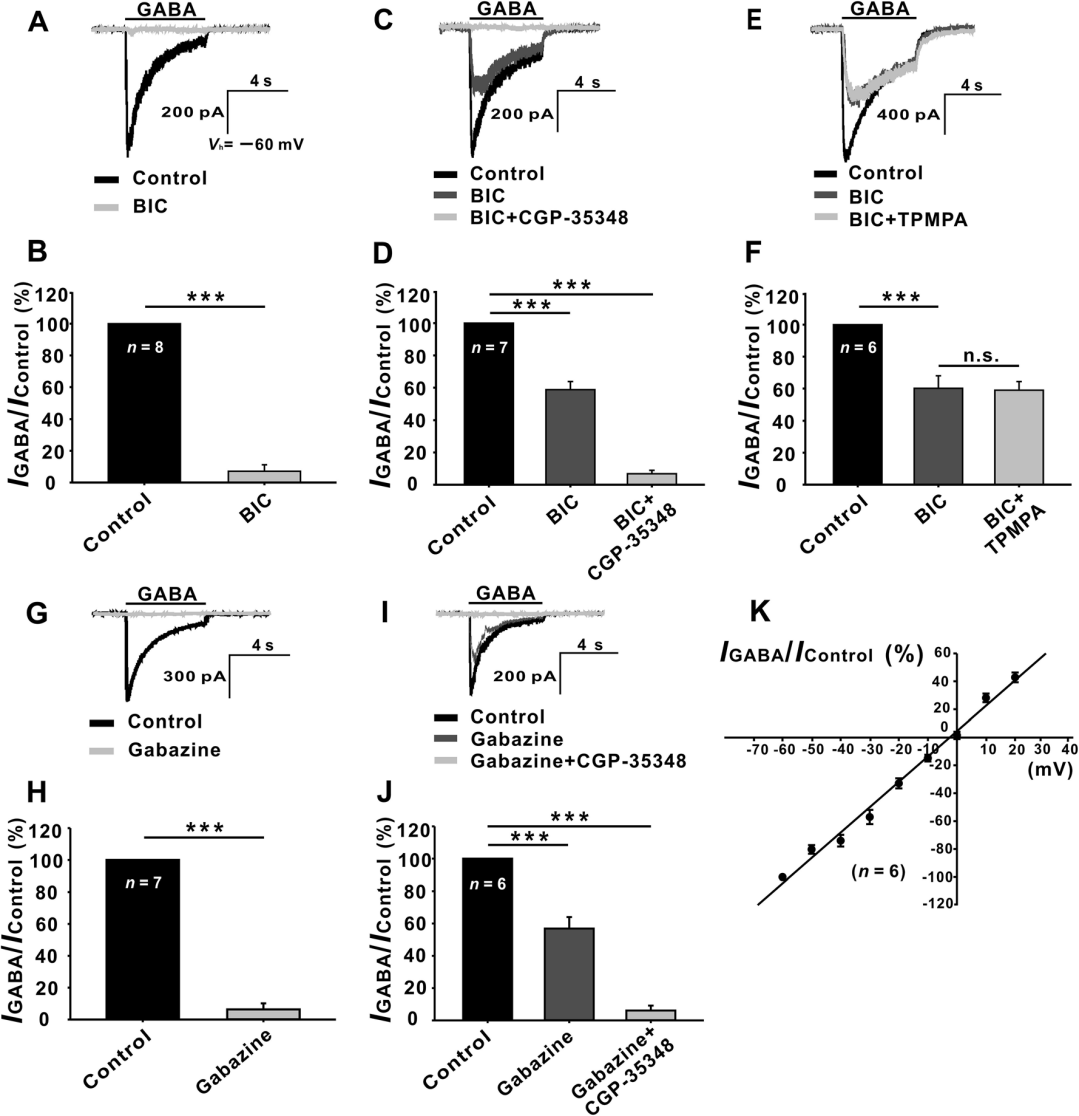
Characterization of GABA-induced currents in isolated rat retinal GCs. **A** Representative recordings showing that the current induced in a GC by 30 μmol/L GABA is almost completely suppressed by 10 μmol/L bicuculline (BIC). The cell is clamped at *V*_h_ = −60 mV and 30 μmol/L GABA is repetitively applied for 5 s at intervals of 2 min. **C** Representative recordings from another GC, showing that the GABA-induced current is partially attenuated by BIC, and the remaining current is completely eliminated by co-application of 100 μmol/L CGP-35348. **E** Current traces of a GC showing that the GABA-induced current is partially suppressed by BIC, and the remaining current is not changed by co-application of 10 μmol/L TPMPA. **G** Representative recordings showing that the GABA-induced current of a GC is almost completely suppressed by 10 μmol/L gabazine. **I** Representative recordings of another GC showing that the GABA-induced current is partially attenuated by gabazine, and the remaining current is completely eliminated by co-application of 100 μmol/L CGP-35348. **B, D, F, H, J** Bar charts showing statistical analysis of the above data. ***P <0.001, n.s., *P* >0.05, paired Student’s *t*-test. The data are presented as the mean ± SEM in all figures. The data for each cell are normalized to the current amplitude of the cell in normal Ringer’s (control) and then averaged. **K** Average current-voltage relationship of GABA_A_ receptor-mediated currents from six GCs. Current responses for each cell at different holding potentials are normalized to the response obtained at –60 mV. Cell numbers (*n*) are marked inside the bars, and the cell numbers in different bars in the same subgraph are the same. It is also the case for other figures.

The currents induced by GABA were recorded from rhodamine-labeled rat retinal GCs with relatively large somata (>15 μm in diameter) in normal Ringer’s. In some GCs, the currents induced by GABA (30 μmol/L) were almost completely suppressed (7.09% ± 4.12% of control, *P* <0.001, *n* = 8) by 10 μmol/L bicuculline (BIC), an antagonist of GABA_A_ receptors (Fig. 1A, B), which suggests that the GABA currents of these cells are exclusively mediated by GABA_A_ receptors. However, in the remaining GCs, the GABA-induced currents were reduced to 58.76% ± 5.02% of control (*P* <0.001, *n* = 7) (Fig. 1C, D) in the presence of 10 μmol/L BIC, and the remaining currents were almost eliminated by perfusing CGP-35348 (100 μmol/L), an antagonist of GABA_B_ receptors (6.79% ± 2.14% of control, *P* <0.001, *n* = 7) (Fig. 1D). Moreover, in the cells in which BIC only partially suppressed the GABA currents (60.09% ± 7.86% of control, *P* <0.001, *n* = 6) (Fig. 1E, F), co-application of BIC and the GABA_C_ receptor antagonist TPMPA (10 μmol/L) did not further suppress the GABA currents (58.94% ± 5.43% of control, *P* <0.001,*n* = 6). Similar results were obtained with the application of gabazine, another GABA_A_ receptor antagonist. That is, in some GCs, addition of 10 μmol/L gabazine almost completely suppressed the GABA currents (6.34% ± 3.79% of control, *P* <0.001,*n* = 7) (Fig. 1G, H), while in the remaining GCs, 10 μmol/L gabazine suppressed the GABA currents to 56.89% ± 7.12% of control (*P* <0.001,*n* = 6) (Fig. 1I, J), and co-application of 100 μmol/L CGP-35348 almost eliminated the currents (6.12% ± 3.05% of control, *P* <0.001, *n* = 6) (Fig. 1I, J). The current-voltage relationship of the GABA_A_ receptor-mediated currents was linear, with a reversal potential of −3.1 ± 2.3 mV (*n* = 6, Fig. 1K), which was very close to the E_Cl_^-^ (−4.3 mV) calculated according to the Nernst equation. These results indicated that the GABA currents of these GCs are mediated by both GABA_A_ and GABA_B_ receptors. All these data suggest that GCs express functional GABA_A_ and GABA_B_ receptors, but not GABAc receptors, and this is consistent with the results of *in situ* hybridization and immunohistochemistry showing that GCs express GABA_A_ and GABA_B_ receptors [23–27].

### Exendin-4 Suppresses GABA Currents in GCs

**Fig. 2:**
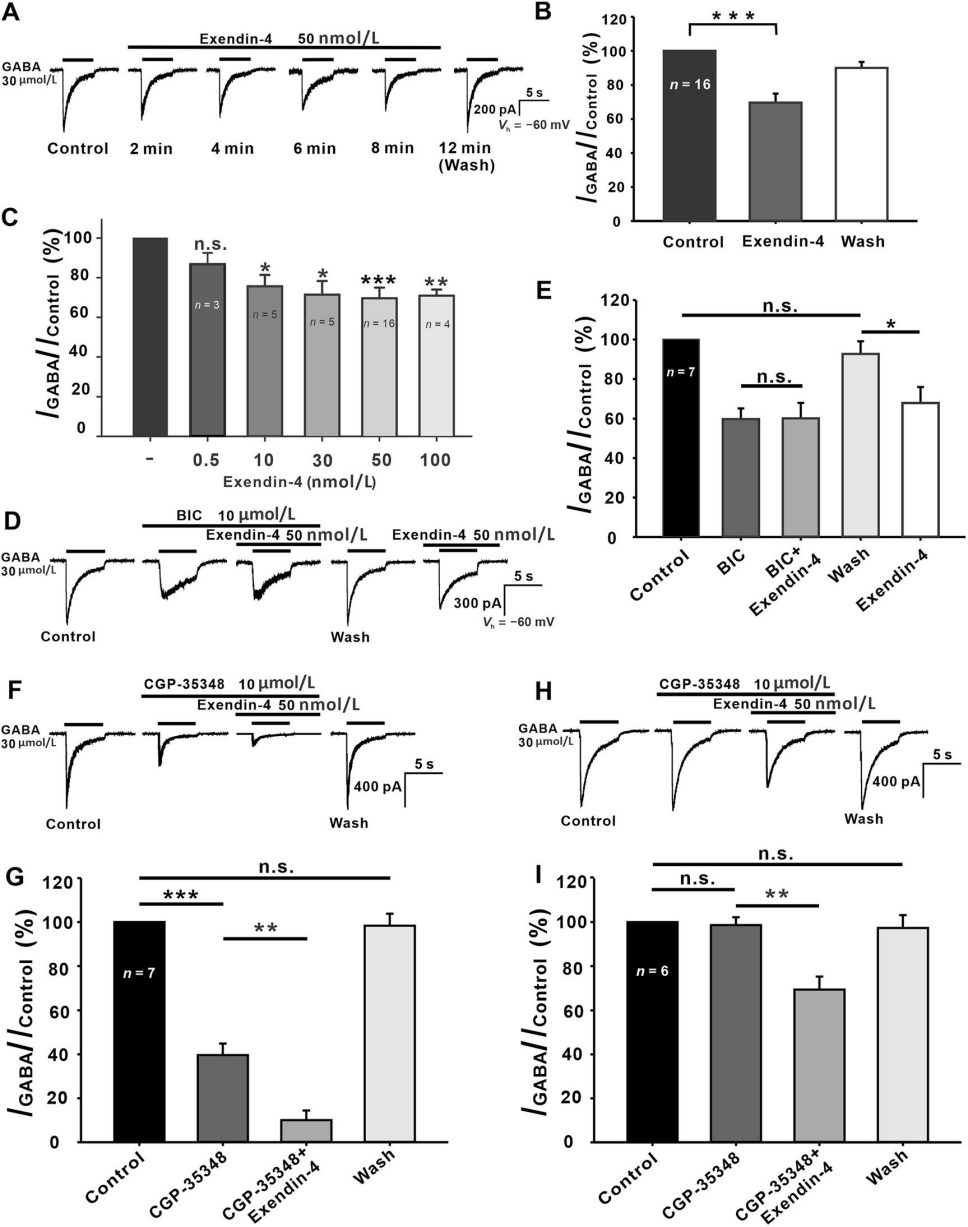
Effects of exendin-4 on GABA currents in isolated rat GCs. **A** Representative recordings showing the effect of 50 nmol/L exendin-4 on GABA currents in a GC. Drug application is indicated by the horizontal lines above the current traces and the times at which the current traces were recorded are marked below (min). **B** Bar chart summarizing the effects of exendin-4 on GABA current amplitude. **C** Exendin-4 suppresses GABA currents in a dose-dependent manner. **D** Representative recordings of a GC showing that GABA currents are largely suppressed by 10 μmol/L BIC. The remaining current (GABA_B_ receptor-mediated current) is not further suppressed by 50 nmol/L exendin-4. After the response returns to the control level on washout, exendin-4 addition suppresses the GABA current. **E** Bar chart summarizing the effects of BIC and exendin-4 on the GABA currents. **F** Representative recordings of a GC showing that GABA current is largely suppressed by 100 μmol/L CGP-35348. The remaining current (GABA_A_ receptor-mediated current) is further suppressed by exendin-4. **G** Bar chart summarizing the effects of CGP-35348 and exendin-4 on the GABA current. **H** Representative recordings of another GC showing that the peak amplitude of GABA current is hardly changed by CGP-35348, and then exendin-4 addition still suppresses the GABA current. **I** Bar chart summarizing the effects of CGP-35348 and exendin-4 on GABA currents. *P <0.05, **P <0.01, ***P <0.001, n.s., *P* >0.05, one-way ANOVA with *post hoc* Tukey’s test.

The endogenous GLP-1 is highly sensitive to degradation by dipeptidyl peptidase IV (DPP-IV) [28–32]; for this reason, we investigated the effects of the protease-resistant long-acting GLP-1R agonist exendin-4 [28, 33, 34] on the GABA currents of GCs (Fig. 2A). No detectable current in GCs was elicited by perfusion of 50 nmol/L exendin-4 (data not shown). After applying exendin-4 for ~2 min, the amplitude of peak current decreased and tended to stabilize in ~6 min (Fig. 2A). The current returned to the control level following a 4-min washout. Suppression by exendin-4 of GABA currents was recorded in most of the tested GCs (16 out of 18, 88.9%). On average, the current amplitudes of these cells after 6 min incubation with 50 nmol/L exendin-4 were decreased to 69.08% ± 5.71% of control (*P* <0.001, *n* = 16, Fig. 2B). In the other two cells, exendin-4 had no effect on the GABA currents (2/18, 11.1%). When the significance test was performed on the whole data set (18 cells), exendin-4 inhibited the GABA currents to a smaller extent (76.96% ± 4.41% of control), but the difference was still statistically significant (*P* <0.01 *vs* control).

We further investigated the concentration-dependence of the exendin-4 effects on GABA currents. The GABA current amplitude recorded after 6 min incubation with exendin-4 was normalized to the level recorded before the incubation (control). As shown in Fig. 2C, 0.5 nmol/L exendin-4 did not significantly suppress the GABA currents (89.81% ± 6.26% of control, *P* >0.05, *n* = 3), but when the exendin-4 concentration was increased to 10, 30, 50, and 100 nmol/L, the GABA currents were suppressed to 74.35% ± 6.79% (*P* <0.05, *n* = 5), 71.92% ± 8.34% (*P* <0.05, *n* = 5), 69.08% ± 5.71% (*P* <0.001, *n* = 16), and 70.66% ± 3.24% (*P* <0.01, *n* = 4) of control, respectively. Based on these data, we chose 50 nmol/L exendin-4 for subsequent experiments, unless otherwise specified.

Since GABA currents were mediated by both GABA_A_ and GABA_B_ receptors in ~62% of GCs, whether exendin-4 regulates GABA_A_ and GABA_B_ currents differentially was further investigated. Application of BIC (10 μmol/L) greatly inhibited the GABA_A_ current (the initial transient component in response to GABA) in some GCs and isolated the GABA_B_ current (the fairly sustained current) (Fig. 2D). The peak amplitudes of GABA_B_ currents were hardly changed by exendin-4 (59.76% ± 5.42% of control for BIC *vs* 60.14% ± 7.86% of control for BIC + exendin-4, *P* >0.05,*n* = 7) (Fig. 2D, E). The response returned to the control level after 6 min washout, and then perfusion of exendin-4 still suppressed the GABA currents to 67.89% ± 8.14% of control (*P* <0.05 *vs* control). Furthermore, in the GCs that expressed both GABA_A_ and GABA_B_ receptors, perfusion with 100 μmol/L CGP-35348 greatly inhibited the GABA_B_ currents (39.63% ± 5.23% of control, *P* <0.001, *n* = 7) (Fig. 2F, G), and the remaining GABA_A_ currents were further suppressed by co-application of exendin-4 (39.63% ± 5.23% of control for CGP-35348 *vs* 10.05% ± 4.39% of control for CGP-35348 + exendin-4, *P* <0.01) (Fig. 2F, G). In other GCs that only expressed GABA_A_ receptors, perfusion with 100 μmol/L CGP-35348 did not change the GABA currents, and addition of exendin-4 significantly inhibited them (98.63% ± 3.57% of control for CGP-35348 *vs* 69.31% ± 5.94% of control for CGP-35348 + exendin-4, *P* <0.01, *n* = 6) (Fig. 2H, I). All these results indicate that exendin-4 suppresses the GABA_A_ receptor-, but not the GABA_B_ receptor-mediated currents of GCs.

### GLP-1R Mediates Exendin-4-/GLP-1-induced Suppression of GABA Currents in GCs

**Fig. 3:**
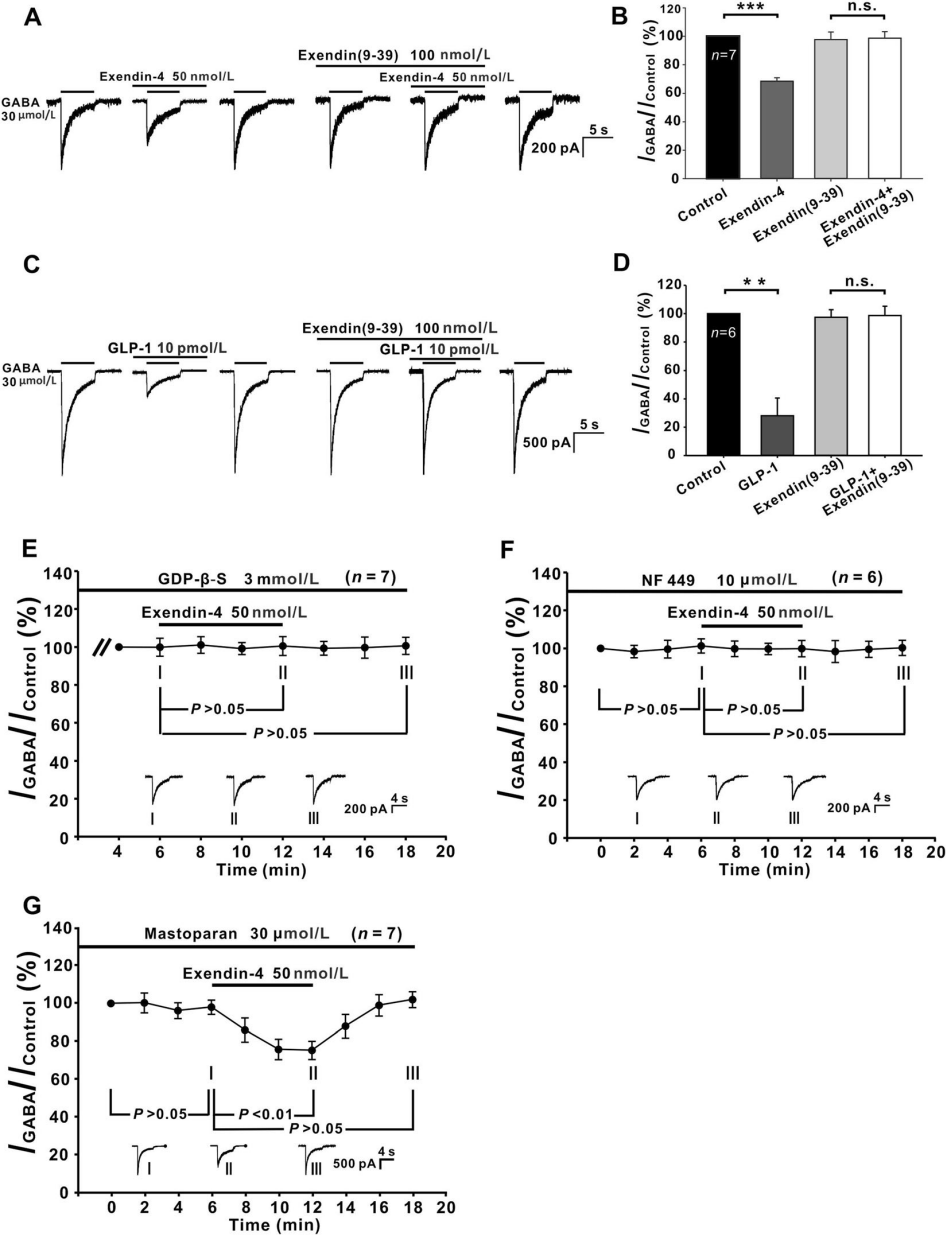
Exendin-4-/GLP-1-induced suppression of GABA currents in GCs is mediated by G_s_-linked GLP-1R. **A** Representative current traces from a GC showing the effects of exendin-4 (50 nmol/L) on the GABA current amplitude in Ringer’s, and in the presence of 100 nmol/L exendin(9-39). **B** Bar chart summarizing the changes in GABA current amplitudes caused by exendin-4 alone and in the presence of exendin(9-39). **C** Current traces of another GC showing the effects of GLP-1 (10 pmol/L) on the GABA current amplitude in Ringer’s, and in the presence of 100 nmol/L exendin(9-39). **D** Bar chart summarizing the changes in GABA current amplitudes caused by GLP-1 alone and in the presence of exendin(9-39). **P <0.01, ***P <0.001. **E** Changes in GABA currents caused by exendin-4 plotted as a function of time during internal infusion of 3 mmol/L GDP-β-S. Note that during the infusion, the addition of exendin-4 does not change the current amplitudes. **F** Averaged time course of the effect of exendin-4 on GABA currents in NF 449-treated GCs. Exendin-4 does not change the currents in the NF 449-treated GCs. **G** Effects of exendin-4 on GABA currents plotted as a function of time during internal infusion of 30 μmol/L mastoparan. Mastoparan *per se* has no effect on the GABA currents of GCs, and co-application of exendin-4 persists in significantly suppressing the GABA currents. The waveforms shown below the data lines are the current responses recorded at the times indicated by I, II, and III shown in **E**, **F,** and **G**. **P <0.01, ***P <0.001, n.s., *P* >0.05, one-way ANOVA with *post hoc* Tukey’s test.

It has been shown that GLP-1R is expressed on neurons of the GCL in the rat retina [10, 11], so we further investigated whether GLP-1R mediated the exendin-4-induced inhibition of GABA currents in GCs. A representative result is shown in Fig. 3A. As a general rule, we tested the effect of exendin-4 on a GC before an experiment to ensure that its GABA current was indeed inhibited by exendin-4. When the current returned to the control level after washout, 100 nmol/L exendin(9-39), a competitive GLP-1R antagonist [35], was perfused for 6 min. Exendin(9-39) alone had no effect on the GABA currents (97.7% ± 5.94% of the control, *P* >0.05 *vs* control, *n* = 7) (Fig. 3A, B), and in the presence of exendin(9-39), addition of exendin-4 for 6 min did not affect the currents [98.63% ± 5.71% of control, *P* >0.05 *vs* exendin(9-39)] (Fig. 3B).

We also determined whether GLP-1 had effects similar to exendin-4. Since the maximal concentration of GLP-1 in human postprandial plasma is <40 pmol/L [1], we chose 10 pmol/L GLP-1 for extracellular perfusion. As shown in Fig. 3C and D, application of GLP-1 for 6 min significantly suppressed the GABA currents of GCs to 28.11% ± 12.50% of control (*P* <0.01, *n* = 6), and the extent of suppression was larger than that of exendin-4 (69.08% ± 5.71% of control). The currents returned to the control level following a 2-min washout. Moreover, the GLP-1-induced suppression was also abolished by exendin(9-39). These results indicate that both exendin-4 and GLP-1 suppress GABA currents by activating GLP-1Rs.

Exendin-4 has been widely used in the treatment of diabetes because its plasma half-life (120 min) is much longer than GLP-1 (1.5 min) [36, 37]. Therefore, we selected exendin-4 for further mechanism study. Because GLP-1R is a G-protein-coupled receptor [38], to determine whether G-protein is associated with the suppression of GABA currents by exendin-4, we added GDP-β-S, a nonhydrolyzable G-protein inhibitor, into patch pipettes. After whole-cell recording from a GC, we waited 4 min to allow GDP-β-S to diffuse throughout the cell. When the GABA currents of GCs reached a stable level at ~6 min after membrane rupture (control), application of exendin-4 for 6 min failed to suppress the currents (99.35% ± 3.64% of control, *P* >0.05, *n* = 7) (Fig. 3E). Since GLP-1R can be coupled to G_s_ or G_i/o_ [39–41], we further investigated which subtype(s) of G-proteins may be associated with the exendin-4 effect. Cell suspensions were preincubated with 10 μmol/L NF 449, a G_s_ antagonist, for at least 30 min before recording and then exposure to exendin-4 for 6 min failed to change the GABA currents of these cells (99.87% ± 4.31% of control) (*P* >0.05, *n* = 6) (Fig. 3F). Furthermore, internal infusion of 30 μmol/L mastoparan, a peptide activator of G_i_ and G_o_, for 6 min did not change the GABA currents of GCs (control), and in the presence of mastoparan, applying exendin-4 for 6 min still suppressed the GABA currents to 76.85% ± 4.54% of control (*P* <0.01, *n* = 7) ( Fig. 3G). These results suggest that GLP-1R is coupled to G_s_ in rat GCs.

### cAMP-PKA Signaling Pathway Mediates Exendin-4-induced Suppression of GABA Currents

**Fig. 4:**
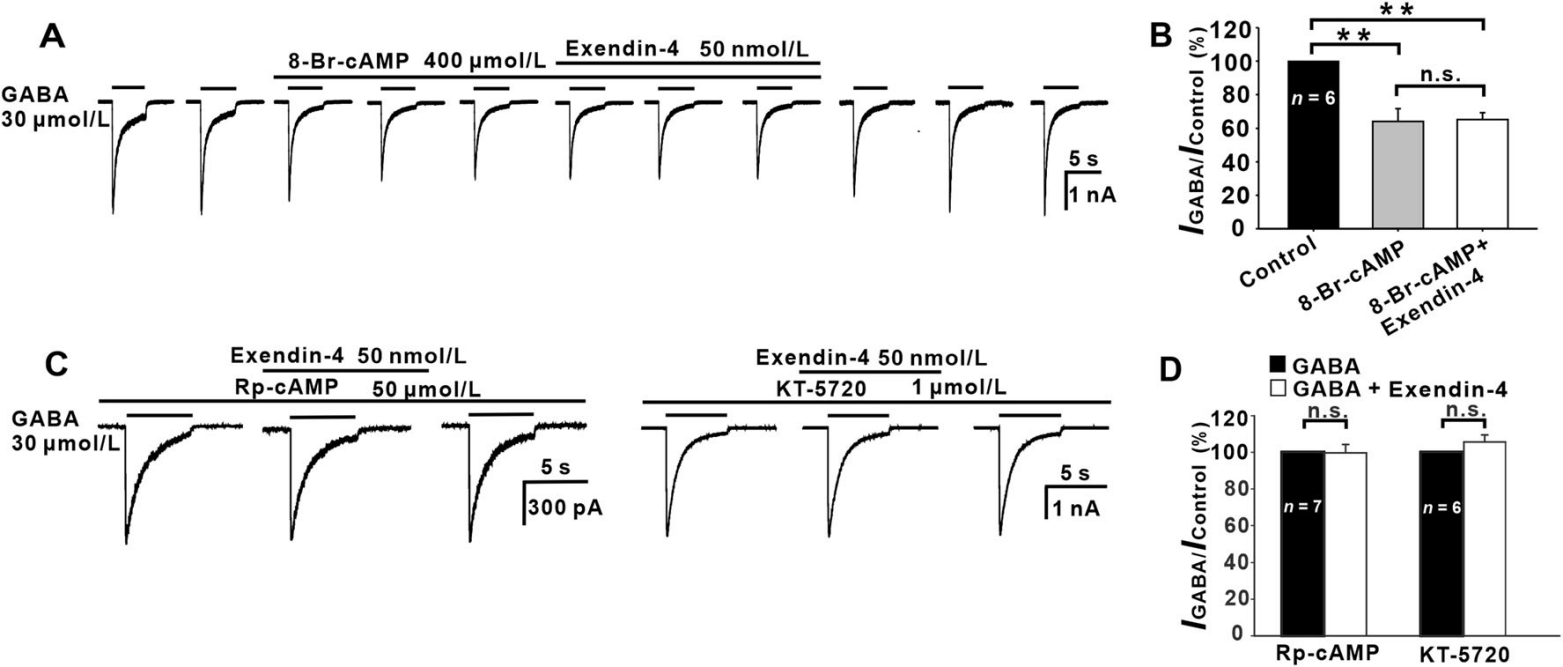
Involvement of the cAMP-PKA pathway in the exendin-4-induced suppression of GABA currents. **A** Representative recordings of a GC showing that perfusion with 400 μmol/L 8-Br-cAMP reduces the GABA currents. In the presence of 8-Br-cAMP, exendin-4 no longer suppresses the currents. **B** Bar chart summarizing the effects of 8-Br-cAMP and exendin-4 on the GABA current amplitudes. **P <0.01. **C** Representative recordings from two GCs, showing that internal infusion of 50 μmol/L Rp-cAMP (left) or 1 μmol/L KT-5720 (right) eliminates the exendin-4-induced reduction of the GABA currents. **D** Bar chart summarizing the effects of Rp-cAMP and KT-5720 on GABA currents. *P* <0.01, n.s., *P* >0.05, one-way ANOVA with *post hoc* Tukey’s test.

Following activation of GLP-1Rs, the main intracellular signaling pathway stimulates G_s_, which in turn activates adenylate cyclase, resulting in increased intracellular cAMP levels and activation of PKA [42, 43]. To investigate whether this pathway is involved, we studied the effects of extracellular perfusion of 8-Br-cAMP, a membrane-permeable cAMP analog, on the GABA currents of GCs. Fig. 4A shows that GABA currents of a GC were gradually suppressed by applying 8-Br-cAMP (400 μmol/L) and reached a stable level 6 min (control) after 8-Br-cAMP application. Co-application of exendin-4 did not change the suppression of the peak currents. Average data showed that the peak current amplitude was suppressed to 64.18% ± 7.42% of control (*P* < 0.01,*n* = 6) (Fig. 4B) by 8-Br-cAMP and applying exendin-4 did not cause further suppression (65.32% ± 3.83% of control, *P* < 0.01 *vs* control and *P* > 0.05 *vs* 8-Br-cAMP) (Fig. 4B). The action of 8-Br-cAMP was reversible. Furthermore, after intracellular application of the PKA inhibitor Rp-cAMP (50 μmol/L), exendin-4 addition no longer changed the currents (98.57% ± 5.01% of control, *P* > 0.05,*n* = 7) (Fig. 4C, D). In addition, when another PKA inhibitor, KT-5720, was intracellularly applied to GCs, additional exendin-4 also failed to affect the GABA currents (102.86% ± 4.34% of control, *P* >0.05, *n* = 6) (Fig. 4C, D).

### Involvement of Ca^2+^ and CaM in Exendin-4-induced Suppression of GABA Currents

**Fig. 5:**
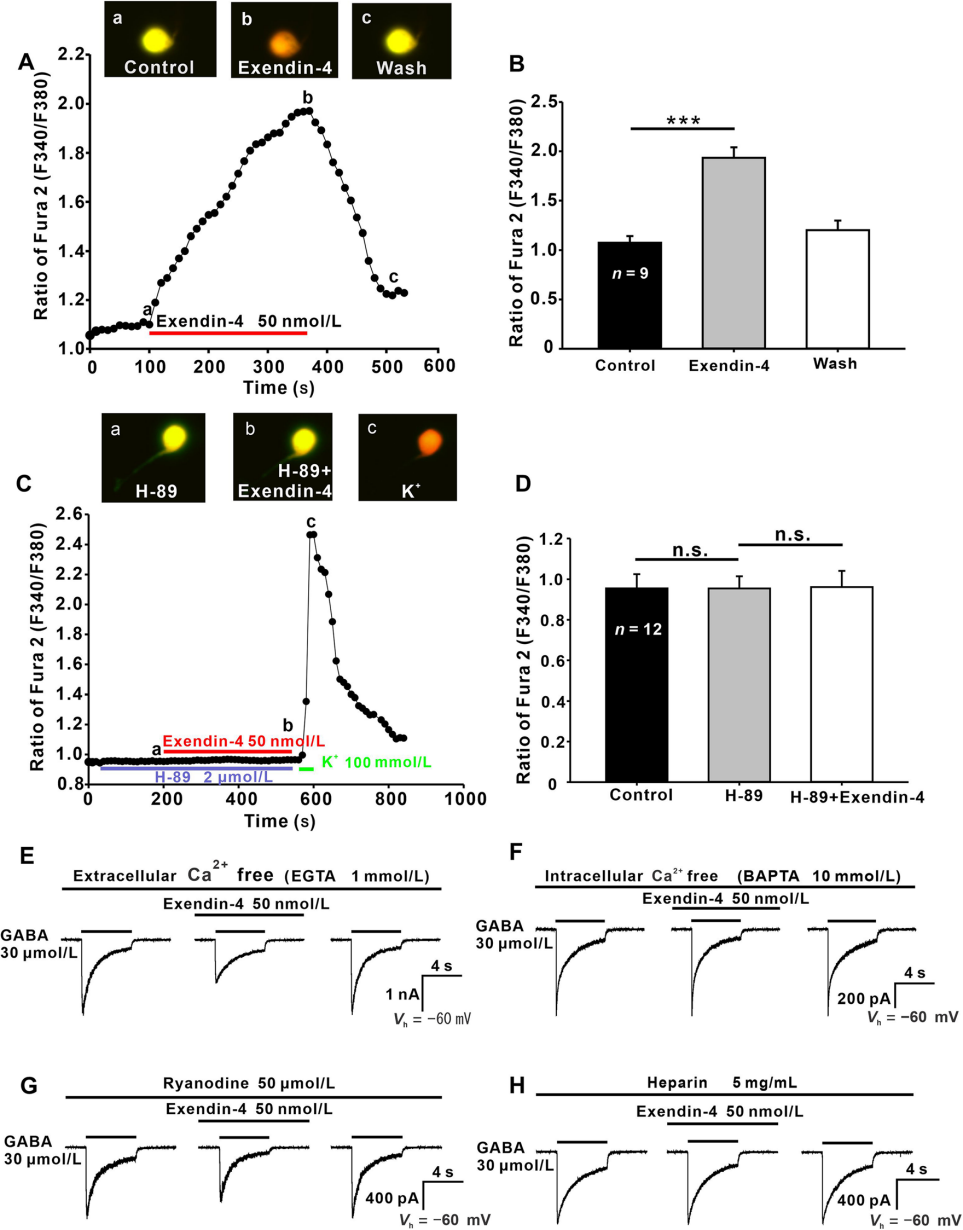
Intracellular Ca^2+^ relevance of exendin-4-induced suppression of GABA currents in GCs. **A** Fura-2 Ca^2+^ recordings from a GC, represented by the F340/F380 ratio, showing the addition of 50 nmol/L exendin-4 dramatically increases [Ca^2+^]_i_ in a reversible manner. Three CCD images of a GC loaded with Fura-2 were captured at times indicated by a, b, and c in the data line. **B** Bar chart summarizing the changes in [Ca^2+^]_i_ induced by exendin-4 in GCs. ***P <0.001. **C** A continuous recording of [Ca^2+^]_i_ in a GC showing that 50 nmol/L exendin-4 fails to change [Ca^2+^]_i_ in the presence of 2 μmol/L H-89, but a brief application of 100 mmol/L KCl induces a dramatic increase in [Ca^2+^]_i_. Three CCD images of the GC loaded with Fura-2 AM were captured at the times indicated by a, b, and c in the data line. **D** Bar chart summarizing the changes in [Ca^2+^]_i_ induced by H-89 and exendin-4. **E** Representative recordings from a GC showing that eliminating extracellular Ca^2+^ with 1 mmol/L EGTA does not affect the action of exendin-4 in reducing GABA currents. **F** Representative recordings from a GC showing that eliminating intracellular Ca^2+^ with the internal infusion of 10 mmol/L BAPTA completely blocks the exendin-4-induced suppression of GABA currents. **G** Depleting ryanodine-sensitive intracellular stores with ryanodine (50 μmol/L) infusion does not affect the exendin-4-induced suppression of GABA currents. **H** Internal infusion of heparin (5 mg/mL) completely blocks the actions of exendin-4. ***P <0.001, n.s., *P* >0.05, one-way ANOVA with *post hoc* Tukey’s test.

Activation of PKA causes an increase in [Ca^2+^]_i_ by regulating Ca^2+^ release from intracellular stores [44, 45]. Moreover, there is much evidence showing that GABAR activity is modulated by [Ca^2+^]_i_ [46–51]. We used Ca^2+^ imaging with Fura-2 to test whether exendin-4 could change [Ca^2+^]_i_ in dissociated GCs. As illustrated by an example in Fig. 5A, the application of 50 nmol/L exendin-4 caused a gradual increase in [Ca^2+^]_i_ of the GC in a reversible manner, the F340/F380 ratio reaching a maximum in 4 min. Exendin-4-induced changes in [Ca^2+^]_i_ of the GC soma are shown in three CCD images (Fig. 5A a−c). In the nine GCs tested, the mean peak ratio of Fura-2 (F340/F380) after exendin-4 perfusion was 1.93 ± 0.11, which was significantly higher than that in Ringer’s (1.08 ± 0.07, *P* <0.001) (Fig. 5B).

To investigate whether the increase of [Ca^2+^]_i_ in the GCs is related to the PKA activation induced by exendin-4, the changes of [Ca^2+^]_i_ were measured by Ca^2+^ imaging when PKA activity was inhibited by H-89. There was no change in [Ca^2+^]_i_ in GCs, either when perfused H-89 alone (0.95 ± 0.06 *vs* 0.96 ± 0.07 for control, *n* = 12) or along with exendin-4 (0.96 ± 0.08 *vs* 0.96 ± 0.07 for control, *n* = 12) (all *P* >0.05) (Fig. 5C, D). The data indicate that the exendin-4-induced [Ca^2+^]_i_ elevation may be a result of PKA activation.

There are two sources for increased [Ca^2+^]_i_: the influx of extracellular Ca^2+^ through voltage-gated Ca^2+^ channels on the plasma membrane, and intracellular Ca^2+^ pools. When GCs were perfused by Ca^2+^-free solution containing the Ca^2+^ chelator EGTA (1 mmol/L) [18], exendin-4 still decreased the GABA currents to 67.33% ± 5.16% of control (*P* <0.001, *n* = 6) (Fig. 5E), indicating that the exendin-4 effect was not associated with changes in extracellular Ca^2+^ levels under our experimental conditions. In contrast, when GCs were intracellularly dialyzed with Ca^2+^-free solution containing the Ca^2+^ chelator BAPTA (10 mmol/L) [52], exendin-4 application did not inhibit the GABA currents (99.04% ± 4.01% of control, *P* >0.05, *n* = 5) (Fig. 5F), suggesting that intracellular Ca^2+^ is indeed associated with the exendin-4 effect.

IP_3_- and/or ryanodine-sensitive pathways mediate the release of Ca^2+^ from intracellular Ca^2+^ pools. After intracellular infusion of 50 μm ryanodine to deplete the ryanodine-sensitive Ca^2+^ sites [53], exendin-4 continued to significantly suppressed the GABA currents of GCs (70.67% ± 4.14% with ryanodine alone, *P* <0.001, *n* = 7) ( Fig. 5G). In contrast, during internal infusion of heparin (5 mg/mL), an IP_3_ receptor antagonist, additional exendin-4 did not further suppressed the GABA currents of GCs (96.65% ± 2.13% of heparin alone, *P* >0.05, *n* = 5) (Fig. 5H).

**Fig. 6:**
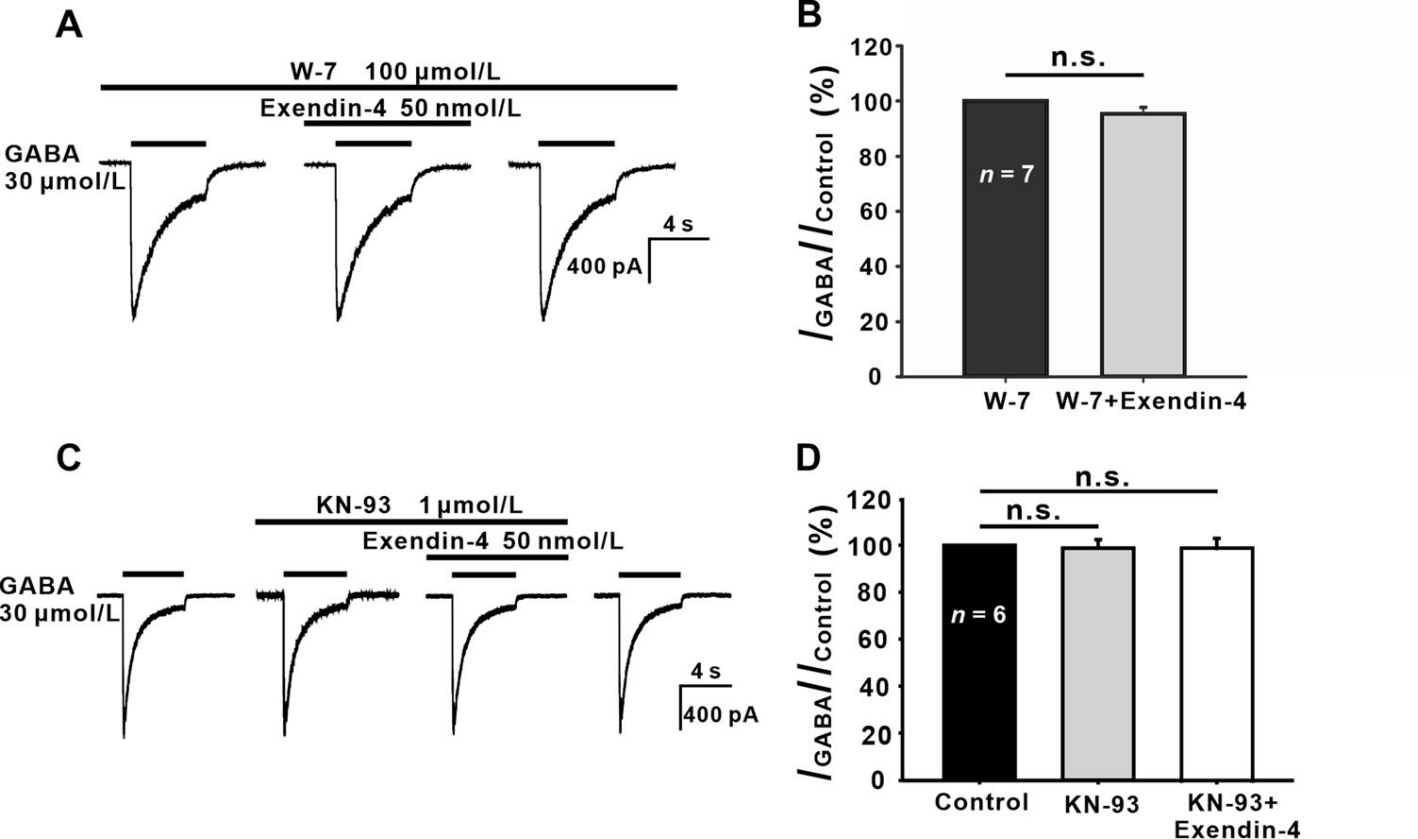
Involvement of CaM and CaMKII in the exendin-4-induced suppression of GABA currents. **A**, **C** Representative recordings from two GCs, showing that exendin-4 (50 nmol/L) no longer suppresses the GABA currents during the internal infusion of 100 μmol/L W-7 (**A**) or extracellular perfusion with 1 μmol/L KN-93 (**C**). **B**, **D** Bar charts summarizing the results for the effects of W-7 (**B**) and KN-93 (**D**). n.s., not significant, paired Student’s *t*-test.

Ca^2+^/CaMKII has been found to play prominent roles in orexin A-mediated inhibition of GABA_A_ receptor [54]. We further examined the effect of the CaM blocker W-7 [55], and found during intracellular infusion of 100 μmol/L W-7 (control), exendin-4 did not change the GABA currents of GCs (95.47% ± 3.67% of control, *P* >0.05, *n* = 7) (Fig. 6A, B). Moreover, after perfusion of KN-93, a CaMKII inhibitor, the addition of exendin-4 failed to suppress the GABA currents (97.90% ± 5.01% of control, *P* >0.05, *n* = 6) (Fig. 6C, D). These results suggest that CaM/CaMKII is involved in the exendin-4 effect on GABA currents.

## PI/PC-PLC-Independent Effects of Exendin-4

**Fig. 7:**
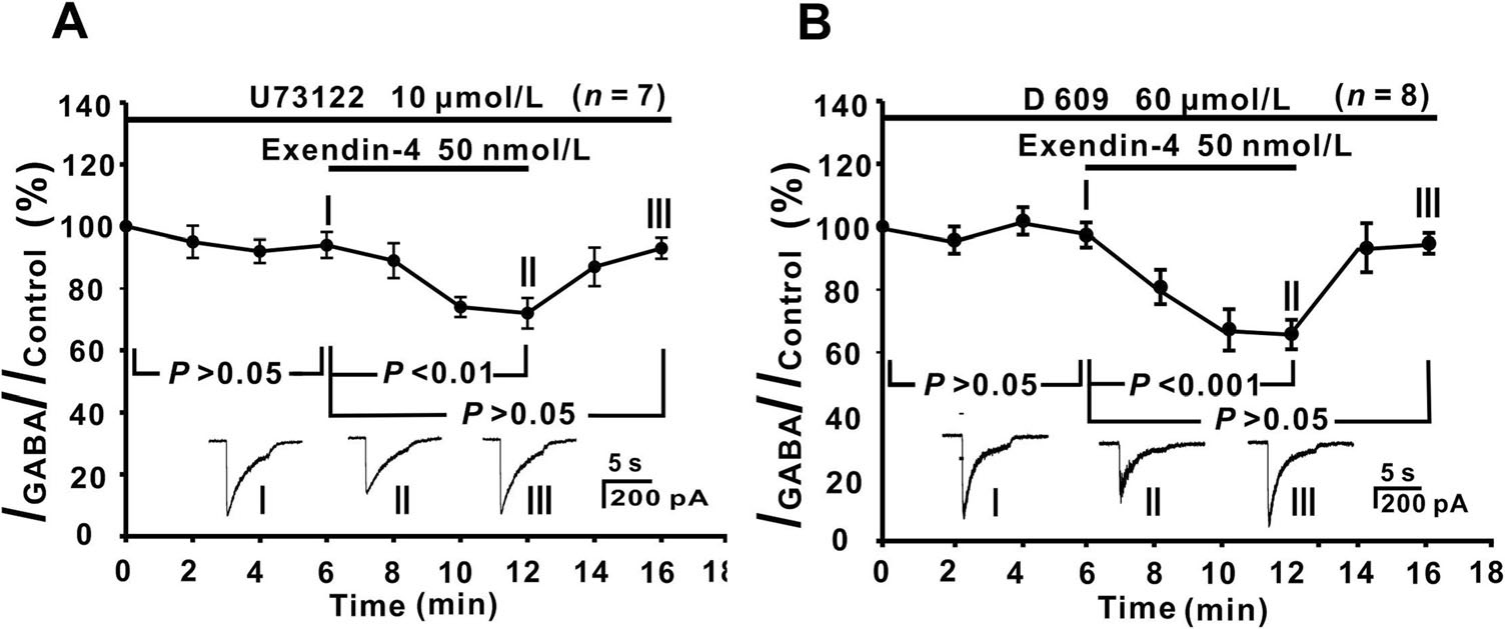
No involvement of the PI-/PC-PLC signaling pathway in the action of exendin-4. **A**, **B** Plots of the average peak current amplitudes as a function of time, showing that exendin-4-induced suppression of GABA currents is still seen in the presence of 10 μmol/L U73122 (**A**) or 60 μmol/L D 609 (**B**). The waveforms shown below the data lines are the current responses recorded at the times indicated by I, II, and III in **A** and **B**. Note that exendin-4 has similar suppressive effects on the GABA currents compared with normal Ringer’s. *P* <0.01, *P* <0.001, *P* >0.05, one-way ANOVA with *post hoc* Tukey’s test.

While GLP-1 has been shown to regulate the phosphatidylinositol-phospholipase C (PI-PLC) or phosphatidylcholine-phospholipase C (PC-PLC) activity in mouse pancreatic β cells [56], experiments with the PI-PLC inhibitor U73122 or with the PC-PLC inhibitor D609, did not support the involvement of these two pathways in the exendin-4 effects on GCs. As shown in Fig. 7A and B, internal infusion of 10 μmol/L U73122 or 60 μmol/L D609 for 6 min did not change GABA currents of GCs (control), then addition of exendin-4 for 6 min still suppressed the GABA currents (73.28% ± 5.09% of control, *P* <0.01 for U73122, *n* = 7; 67.41% ± 5.43% of control, *P* <0.001 for D609, *n* = 8).

**Fig. 8:**
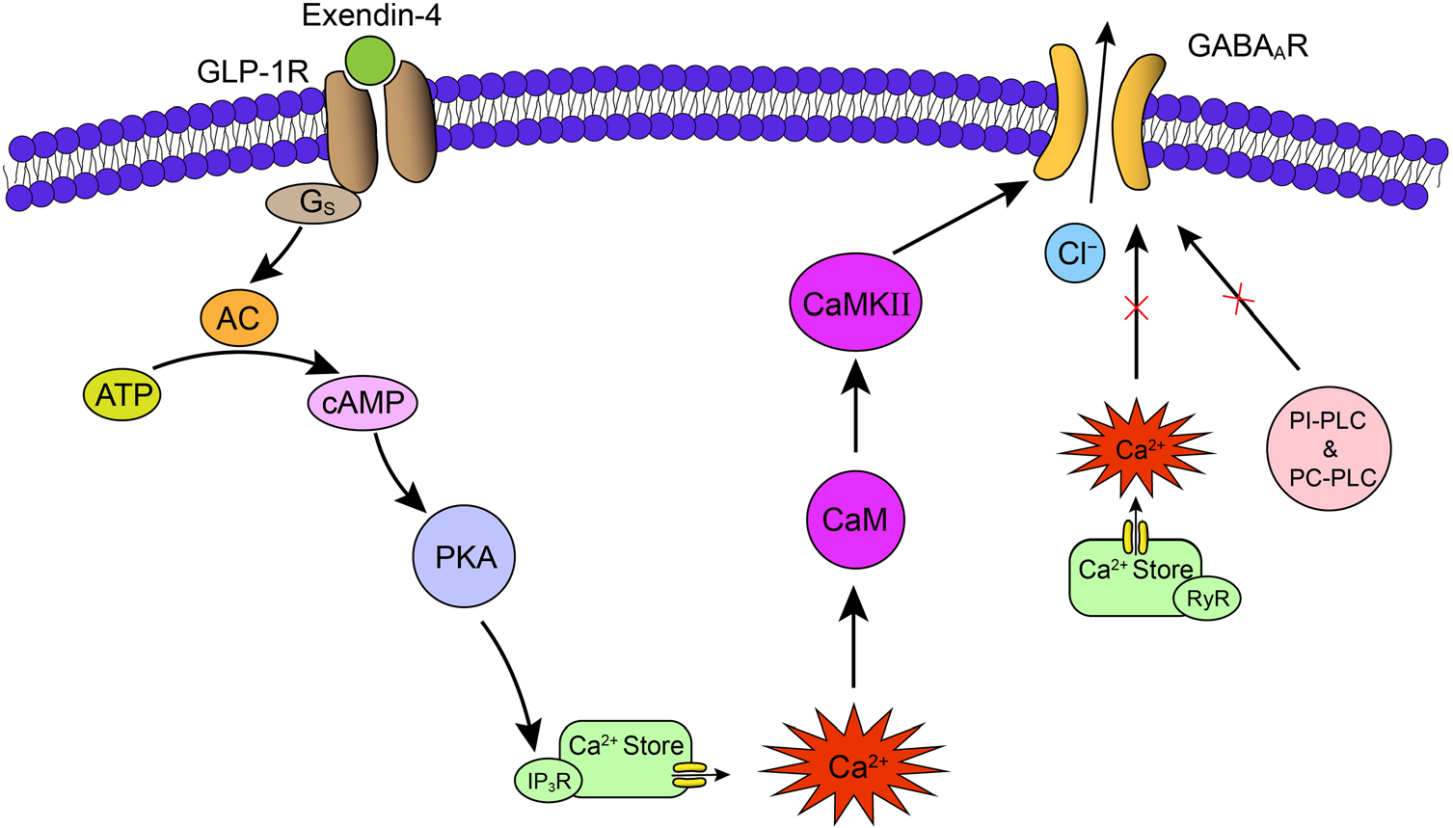
Schematic showing the putative signaling pathway that mediates the suppression of GABA currents by exendin-4 in rat retinal GCs. By activating G_s_-coupled GLP-1Rs, exendin-4 suppresses GABA currents *via* a distinct intracellular cAMP-PKA/IP_3_/Ca^2+^/CaM/CaMKII signaling pathway. Note that neither PI-PLC nor PC-PLC is involved in the effect. AC, adenylate cyclase; IP_3_R, IP_3_ receptor; CaM, calmodulin; CaMKII, calmodulin-dependent protein kinase II; RyR, ryanodine receptor; PI-PLC, phosphatidylinositol-phospholipase C; PC-PLC, phosphatidylcholine-phospholipase C.

Taken together, the putative signaling pathway mediating the effects of exendin-4 on GABA currents of rat GCs is summarized in the schematic diagram in Fig. 8.

### Exendin-4 Suppresses GABAR-mediated L-IPSCs in GCs of Rat Retinal Slices

To further verify the effect of exendin-4 on GABARs on GCs, we examined the effects of exendin-4 on GABAR-mediated L-IPSCs in rat retinal slice preparations. During perfusion with TTX and strychnine (see Methods for details), the GABAR-mediated L-EPSCs of GCs were recorded. As shown in Fig. S1A and S1B, bath application of 100 nmol/L exendin-4 significantly suppressed the GABAR-mediated L-IPSC to 68.38% ± 4.54% of control (*P* <0.001, *n* = 8), and the response returned to the control level after washout (91.02% ± 7.94% of control, *P* >0.05). We also determined whether GLP-1R mediated the exendin-4 effect on GABAR-mediated L-IPSCs in GCs. Perfusion with 100 μmol/L exendin(9-39) did not change the L-IPSCs (95.09% ± 3.57%, *P* >0.05, *n* = 10) (Fig. S1C, D), and the average current peak amplitude after co-application of exendin-4 for 6 min was 95.53% ± 2.76% of that obtained prior to exendin-4 perfusion (*P* >0.05, *n* = 10).

## Discussion

### GLP-1/exendin-4 Suppresses GABA Currents of Rat GCs by Activating GLP-1R

Although GLP-1 and GLP-1R agonists have been reported to regulate AMPA receptors and GABA_A_ receptors in central neurons [14, 57–60], no data concerning the regulation of ligand-gated channels of retinal neurons by GLP-1/GLP-1R agonists were available. A major finding in the present work was that GLP-1/exendin-4 significantly suppressed GABA currents in rat GCs by activating GLP-1R. This result concurs with the report that GLP-1R-immunoreactivity occurs in the neurons of the rat GCL [10–12]. In plasm, the maximal concentration of GLP-1 after a meal is reported to be <40 pmol/L [1, 14]. Interestingly, in our experiments, 10 pmol/L GLP-1 (normal physiological postprandial concentration) suppressed GABA currents (28.11% ± 12.50% of control) more strongly than 50 nmol/L exendin-4 (69.08% ± 5.71% of control). Different from our results, GLP-1/exendin-4 increases GABA_A_R-mediated tonic currents and GABA_A_R-mediated spontaneous IPSC frequency through GLP-1R activation in rat hippocampal CA3 pyramidal neurons [14]. These results suggest that the modulation by GLP-1/exendin-4 of GABA receptors may vary depending on the cell type.

GLP-1 is a hormone released by intestinal L-cells [1]. In the brain, GLP-1 is synthesized by preproglucagon neurons, which are distributed in the nucleus of the solitary tract of the brain stem [2–5]. Real-time quantitative RT-PCR analysis has revealed that GLP-1 mRNA is present in the human retina [7]. Moreover, immunostaining of GLP-1 has been reported in neurons in the GCL of human and mouse retinas [7, 8]. These data suggest that GLP-1 may be also synthesized and released from GCs and/or displaced amacrine cells in the GCL. Previous studies have shown that GLP-1 can cross the blood-brain barrier [43, 61], but whether it can cross the blood-retina barrier remains unknown.

### Intracellular Mechanisms Underlying Exendin-4-induced Suppression of GABA Currents

GLP-1R has been reported to primarily act through G_s_ [2, 43] and has also been shown to couple with the inhibitory G_i/o_ proteins [40]. Using selective pharmacological inhibitors, we found that G_s_, but not G_i/o_, were involved in the exendin-4-induced suppression of GABA currents (Fig. 3 E–G). Actually, studies on rat dorsal root ganglion neurons have shown that activation of G_s_ decreases GABA-induced currents [62].

Even though the PLC/PKC signaling pathway has been shown to be involved in GLP-1-induced insulin secretion in mouse pancreatic β cells [56], this signaling pathway was unlikely involved in the effect of exendin-4 on GABA currents in GCs, due to the persistence of this effect when PI-PLC or PC-PLC was blocked (Fig. 7). In contrast, our evidence suggested that the cAMP-PKA signaling pathway mediates the exendin-4-induced inhibition in GCs. The inhibitory effect of exendin-4 on GABA current was mimicked by perfusion with the cAMP analog 8-Br-cAMP. Moreover, the exendin-4-induced inhibition of GABA currents was eliminated when PKA was inhibited by KT-5720 or Rp-cAMP (Fig. 4). These results suggest that activating G_s_-linked GLP-1R stimulates adenylyl cyclase and increases the cAMP level, which in turn activates PKA. cAMP and PKA are important signaling molecules responsible for GLP-1R-mediated effects, which have been shown in neurons and non-neuronal cells [63–70].

Furthermore, the exendin-4 effect on GABA currents in GCs was Ca^2+^-dependent. Ca^2+^ imaging showed that exendin-4 significantly increased [Ca^2+^]_i_ in GCs (Fig. 5), consistent with the results reported in cultured hippocampal neurons showing that acute application of GLP-1 induces a transient elevation of [Ca^2+^]_i_ [58]. GLP-1 has been shown to regulate voltage-gated Ca^2+^ channels. For example, GLP-1 enhances L-type Ca^2+^ currents through activation of the cAMP-dependent PKA pathway in canine cardiomyocytes [71], whereas when rat hippocampal neurons are pre-incubated with GLP-1 for 24 h, Ca^2+^ currents are significantly decreased [58]. However, when we chelated extracellular Ca^2+^ with EGTA, exendin-4 still suppressed the GABA currents in GCs, suggesting that Ca^2+^ entry through voltage-gated channels was not be involved in the action of exendin-4 under our experimental conditions. In contrast, when [Ca^2+^]_i_ was greatly reduced by internal infusion of Ca^2+^-free solution, exendin-4 no longer suppressed GABA currents (Fig. 5F). Moreover, the exendin-4 effect on GABA currents was blocked when IP_3_ receptors were blocked by heparin. These data indicate that the Ca^2+^ release from IP_3_ receptor-mediated intracellular pools is associated with the exendin-4 effect on GCs. Elevated [Ca^2+^]_i_ in rat GCs may be a result of exendin-4-induced PKA activation, because our experiments demonstrated that exendin-4 no longer caused changes in [Ca^2+^]_i_ when PKA activity was blocked by H-89 (Fig. 5C, D). Actually, PKA activation has been shown to lead to Ca^2+^ release from intracellular storage in heart cells and hepatocytes through the ryanodine and/or IP_3_ receptor pathway [44, 45, 72].

Many cellular Ca^2+^-stimulated signaling cascades utilize the intermediate, CaM. The binding of Ca^2+^ alters the conformation of CaM and increases its affinity to many CaM-binding proteins, including CaMKII [73]. In GCs, the CaM/CaMKII pathway may mediate the exendin-4 effect, because the inhibitory effect by exendin-4 on GABA currents did not occur when CaM and CaMKII were blocked by W-7 and KN-93, respectively (Fig. 6). Consistent with our results, that CaM mediates the suppression of GABA_A_ currents induced by elevated levels of [Ca^2+^]_i_ has been reported in turtle retinal GCs [49] and rat rod bipolar cells [52], while that Ca^2+^/CaMKII mediates orexin-A-induced inhibition of GABA_A_ currents has been reported in HEK293 cells [54].

GABA is one of the main inhibitory neurotransmitters and is predominantly released by wide-field GABAergic amacrine cells in the inner retina. It has been reported that GABAergic amacrine cells contribute to the organization of the GC receptive field [74–76]. The receptive field surround of GCs may be generated by feedforward inhibition directly onto GCs [75] or presynaptically, by GABAergic feedback inhibition from amacrine cells onto bipolar cell axons [77, 78]. Application of GABA suppresses both the spontaneous and light-evoked activity of all GCs and the GABA_A_R antagonist bicuculline potentiates the spontaneous and light-evoked activity of ON-type GCs [79], which suggests that GABA directly inhibits GCs. Light stimulation-induced GABAR-mediated responses on most GCs in this work indicated that these cells receive direct inhibitory inputs from GABAergic amacrine cells. Suppression by exendin-4 of the GABA responses of GCs suggests that exendin-4 weakens inhibitory inputs mediated by GABA, which modulates the receptive field properties of GCs.

## Supplementary Information

Below is the link to the electronic supplementary material.

Supplementary file1 (PDF 389 kb)
