# Supplementary material for: Signaling Mechanism for Modulation by GLP-1 and Exendin-4 of GABA Receptors on Rat Retinal Ganglion Cells
Source: Neurosci Bull. 2022 Mar 12;38(6):622–36. doi: 10.1007/s12264-022-00826-9 (PMC9206055; doi:10.1007/s12264-022-00826-9)
Supplement: Supplementary file 1 — Supplementary file1 (PDF 389 kb) [file 12264_2022_826_MOESM1_ESM.pdf]

Supplementary Figure

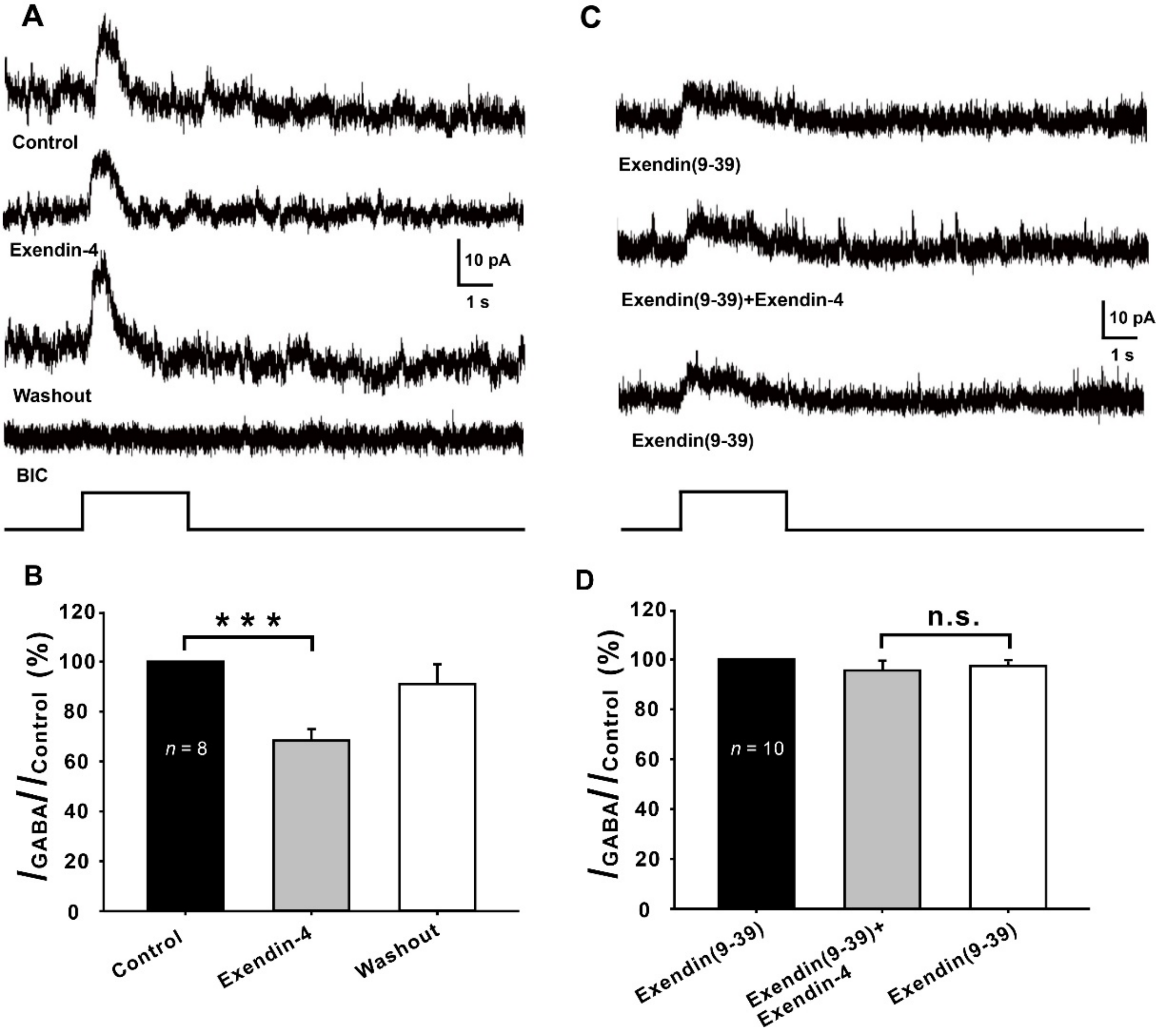

Fig. S1 Exendin-4 suppresses GABAR-mediated L-IPSCs of GCs *via* GLP-1R activation. **A** L-IPSCs from a GC, clamped at 0 mV, elicited by a 3-s, full-field light stimulus in the presence of 1 μmol/L TTX and 1 μmol/L strychnine. Application of 100 nmol/L exendin-4 reversibly suppresses the L-IPSC. The L-IPSC is completely eliminated by 10 μmol/L BIC. **B** Bar chart summarizing the changes of peak amplitudes of GABAR-mediated L-IPSCs induced by exendin-4. ***P <0.001, n.s., P >0.05, one-way ANOVA with *post hoc* Tukey's test. **C** GABAR-mediated L-IPSCs from a GC, showing that in the presence of 100 μmol/L exendin(9-39), application of 100 nmol/L exendin-4 no longer suppresses the current. **D** Bar chart summarizing the changes of peak amplitudes of L-IPSCs induced by exendin(9-39) and co-application of exendin-4. Cell numbers (n) are marked inside the bars, and the cell numbers in different bars in the same subgraph are the same.
